# Influence of Newly Organosolv Lignin-Based Interface Modifier on Mechanical and Thermal Properties, and Enzymatic Degradation of Polylactic Acid/Chitosan Biocomposites

**DOI:** 10.3390/polym13193355

**Published:** 2021-09-30

**Authors:** Faisal Amri Tanjung, Yalun Arifin, Retna Astuti Kuswardani

**Affiliations:** 1 Faculty of Science and Technology, Universitas Medan Area, Medan 20223, North Sumatera, Indonesia; 2 Department of Food Business Technology, Universitas Prasetiya Mulya, BSD Raya Utama, Tangerang 15339, Banten, Indonesia; yalun.arifin@prasetiyamulya.ac.id; 3 Faculty of Agriculture, Universitas Medan Area, Medan 20223, North Sumatera, Indonesia; retna@staff.uma.ac.id

**Keywords:** polylactic acid, chitosan, organosolv lignin, modifying agent, biocomposites

## Abstract

This article aimed to study the effects of chitosan fiber and a newly modifying agent, based on organosolv lignin, on mechanical and thermal performances and the enzymatic degradation of PLA/chitosan biocomposites. A newly modifying agent based on polyacrylic acid-grafted organosolv lignin (PAA-g-OSL) was synthesized via free radical copolymerization using *t*-butyl peroxide as the initiator. The biocomposites were prepared using an internal mixer and the hot-pressed method at various fiber loadings. The results demonstrate that the addition of chitosan fiber into PLA biocomposites remarkably decreases tensile strength and elongation at break. However, it improves the Young’s modulus. The modified biocomposites clearly demonstrat an improvement in tensile strength by approximately 20%, with respect to the unmodified ones, upon the presence of PAA-g-OSL. Moreover, the thermal stability of the modified biocomposites was enhanced significantly, indicating the effectiveness of the thermal protective barrier of the lignin’s aromatic structure belonging to the modifying agent during pyrolysis. In addition, a slower biodegradation rate was exhibited by the modified biocomposites, relative to the unmodified ones, that confirms the positive effects of their improved interfacial interaction, resulting in a decreased area that was degraded through enzyme hydrolysis.

## 1. Introduction

The manufacture of composite materials using ecologically friendly technologies is of great of interest to many academic and industrial practitioners in the areas of polymer science and engineering [1]. This trend is mainly driven by environmental considerations regarding the negative impact of petroleum-derived materials on the environment after their end-use, which are difficult to decompose in a landfill, and also by aiming to attain composite materials that possess the desired properties [2]. Composite materials often contain bio-based polymer. Among the bio-based polymers, polylactic acid (PLA) is the best biopolymer alternative for petro-polymers because of its renewability, biodegradability, biocompatibility, and good thermomechanical performance [3,4]. This biopolymer is derived from plants, such as corn and cassava, and it is known to have a relatively high melting point, strength, and versatility, with performance characteristics similar to synthetic polymers, such as polyethylene terephthalate, polyethylene, etc., [5,6,7].

PLA is regarded as a valuable and important biopolymer that can be utilized to replace synthetic counterparts in many applications, ranging from automotive parts to electronic devices [8,9,10]. However, PLA has some limitations that consequently restrict its widespread application, involving a low thermal resistance, heat distortion temperature, and crystallization rate, whereas other specific properties may be necessary in some different use sectors [11]. As a result, the incorporation of reinforcement agents, such as natural fibers and inorganic substances, represents a broad method for extending and improving the PLA’s performance, particularly with regard to its tensile and thermal properties [12,13,14,15,16]. 

Many natural fibers have been studied in the design of PLA biocomposites that show promising results. Among existing natural fibers, chitosan has demonstrated excellent mechanical and thermal properties that are comparable to cellulose [17]. Chitosan is derived from chitin, which is obtained from the shells of crustaceans, such as crabs, shrimp, and prawns [18]. This natural fiber has been widely used in a variety of scientific applications, including medicine, biotechnology, the textile and food industries, as well as in fiber and plastic applications [19,20,21]. However, the main disadvantages of utilizing it as a reinforcing agent in polymer composites include low dispersion and poor interfacial adhesion, both of which are caused by incompatibility with the hydrophobic matrix polymer. This is demonstrated by the difficulties of the polar hydroxyl groups located on the chitosan surface in building a well-bonded interface with a nonpolar matrix polymer, as strength improvement is dependent on stress transfer at the composite’s interface when an external force is applied [22]. If the interface is poor, the fiber-matrix adhesion will diminish with no enhancement in performance [23]. Consequently, this problem reduces the benefits of using potential reinforcements in polymer composites. 

To address the issue of interaction, the interfacial adhesion in the composite material is chemically altered. Chemical modification has been frequently utilized to enhance the interfacial adhesion in composite systems, since it is an effective technique for reducing the hydrophilic properties of natural fibers. Previous research used treated chitosan fiber as a natural filler in polypropylene composites. The results show that the incorporation of chitosan fiber into the composites increased the tensile modulus and the impact strength, while decreasing the tensile strength significantly [24]. Therefore, the current research is focused on employing chitosan fibers to increase the performances of PLA biocomposites by filler surface modification using a newly developed modifying agent based on grafted organosolv lignin. To the best of our knowledge, investigations involving the utilization of grafted organosolv-lignin-based modifying agents in biocomposites have been less reported in the literature.

Lignin is well-known as a byproduct of the wood pulping process and is one of the abundant vegetal-derived compounds [25]. Because of its complex aromatic structure, which is linked by an ester-bridge, lignin is a highly stable polymer. It has a strong polarity that results from the existence of a huge number of hydroxyl groups, both aliphatic and aromatic [26,27,28]. The lignin used in this study was obtained from lignocellulosic fiber using an established organosolv method with organic solvent and water [29]. The procedure produces lignin with a low molecular weight and a huge number of reaction sites available, making this kind of lignin a suitable surface-modifying agent. However, because of the complexity of its structure, lignin is difficult to dissolve in conventional solvents, causing the limitation of its chemical reactivity [30]. Therefore, a simple copolymerization reaction with acrylic acid was used to improve the lignin reactivity and solubility. The lignin alteration produces a pendant carboxylic moiety, which provides a site for additional reactive reactions.

This research is aimed at studying the effects of chitosan fiber and a newly developed modifying agent based on grafted organosolv lignin on the mechanical and thermal performances of PLA/chitosan biocomposites. Furthermore, the weight loss of the PLA/chitosan biocomposites during enzymatic degradation is examined.

## 2. Materials and Method

### 2.1. Raw Materials

PLA (TT Biotechnology Sdn. Bhd, Penang, Malaysia) had a melt flow index of 5.6 g/10 min (180 °C/2160 g), and a density of 1.27 g·cm^−3^. The chitosan (Hunza Nutriceuticals Sdn Bhd., Parit Buntar, Malaysia) had an average size of 80 µm and a 90% degree of deacetylation (DD). The characteristics of chitosan are listed in Table 1. Commercial-grade diastase (sourced from malt) was supplied by Sigma Aldrich (St. Louis, MO, USA). The ethanol (98%.*v*/*v*), hydrochloric acid, *t*-butyl peroxide, acrylic acid, acetic acid, NaOH, sodium acetate and the sulphuric acid (98%.*v*/*v*) were obtained from Sigma Aldrich and used without further purification. 

### 2.2. Extraction of Organosolv Lignin (OSL) from Lignocellulosic Fiber

The extraction procedure of organosolv lignin (OSL) from lignocellulosic fiber was conducted following the established organosolv method [31]. The fiber was first treated using a mixture of aqueous ethanol and a catalyst (sulphuric acid) at a temperature set of 190 °C for 1 h, with the solid to liquid ratio adjusted at 1:8. The pretreated fiber was then rinsed with aqueous ethanol. The washes were mixed, and 5 vol% of distilled water was added to precipitate the organosolv lignin. The OSL was centrifuged and then dried in an oven at a temperature of 80 °C for 24 h.

### 2.3. Preparation of PAA-grafted OSL

The chemical grafting reaction of OSL with acrylic acid was carried out by utilizing *t*-butyl peroxide as the initiator in a copolymerization reaction. Three grams of lignin sample and *t*-butyl peroxide were mixed with 60 ml of distilled water in the reactor. Acrylic acid was subsequently added slowly into the reaction mixture under constant stirring. The mixture pH was adjusted from around 8.5 to 9.0 by adding 2% NaOH solution. After the reaction was completed, the product was precipitated by adding 6 M HCl. The solid was allowed to stay overnight upon discarding the supernatant. The solid product was rinsed using acidified water (pH = 2–3) prior to drying in an oven.

### 2.4. Chemical Modification of Chitosan

PAA-g-OSL was used to chemically modify the chitosan surface in an ethanol medium. The amount of PAA-g-OSL used was 3% by weight of chitosan fiber. Chitosan fiber was added to the PAA-g-OSL solution, and then vigorously stirred for 4 h. Afterward, the chitosan suspension was filtered and dried for 24 h in an oven at a temperature of 80 °C.

### 2.5. Preparation of PLA/Chitosan Biocomposites

PLA/chitosan biocomposites were prepared in an internal mixer (MCN ELEC Co., Taichung, Taiwan) at 160 °C and 50 rpm. PLA was firstly fed into the mixer to initiate the melt mixing process for 12 minutes. Afterward, chitosan fiber was slowly added, and the compounding was carried out for another 3 min. Next, the compounds were removed and sheeted through a laboratory mill at a 2.0 mm nip setting. The compounds were compression molded at 120 kg·cm^−2^ in an electrically heated hydraulic press. The hot-press procedure consists of three-consecutive steps, involving preheating at 190 °C for 9 min, compression for 3 min at 190 °C, and then cooling pressure for 3 min. A similar procedure was carried out to synthesize the modified PLA/chitosan biocomposites. The formulation of unmodified and modified PLA/chitosan composites is exhibited in Table 2.

### 2.6. Characterization

Tensile tests were conducted using an Instron 5582 machine (Instron, Norwood, MA, USA)according to ASTM D 638-91. A Wallace die cutter was utilized to cut at least five dumbbell specimens of each composition, 1 mm thick, from the molded sheets. The test was conducted at 25 ± 3 °C with a crosshead speed of 20 mm/min. Thermogravimetric (TGA) and derivative thermogravimetric (DTG) analyses were carried out using a TGA Q500 (TA Instruments, New Castle, Germany). The samples were thermally scanned at a temperature range of 30 to 600 °C, at a heating rate of 20 °C/min, with a nitrogen flow of 50 mL/min. Differential scanning calorimetry (DSC) analysis was performed using a DSC Q 1000 (TA Instruments). Samples were scanned at a temperature range of 25 to 250 °C, at a heating rate of 20 °C/min, with a nitrogen flow of 50 ml/min. The instrument software was used to calculate the melting points (T_m_), glass transition temperature (T_g_), crystallization temperature (T_c_), and the enthalpies of the PLA/chitosan biocomposites. The crystallinity of the composites (*X*_c_) was manually calculated using Equation (1):

*X*_c_ (%) = ΔH_f_ × 100/ΔH_f_° (1)

where, ΔH_f_ is the fusion enthalpy of the PLA and biocomposites, and ΔH_f_° is the thermodynamic fusion enthalpy belonging to a fully crystalline PLA (93.6 J/g) [32]. Data from the second heating scan was taken for each sample. An average value from three samples of each specimen was recorded.

A morphological study of the fracture surfaces of PLA/chitosan biocomposites was performed using a scanning electron microscope (SEM), JEOL model JSM 6260 LE. The fracture ends of the specimens were mounted on aluminum stubs and sputter-coated with palladium to avoid electrostatic charging during analysis.

Fourier transform infrared spectroscopy (FTIR) was conducted on unmodified and modified chitosan in ATR mode (Perkin Elmer 1600 Series). Samples were scanned at a resolution of 4 cm^−1^ from 650 to 4000 cm^–1^.

The number of the average molecular weight (M_n_), and weight average molecular weight (M_w_), of the lignin were measured using gel permeation chromatography (GPC) after the acetylation of the lignin to allow dissolution in THF [33]. GPC analysis was carried out using a Perkin Elmer instrument (Waltham, MA, USA) equipped with an interface (PE Series 900).

The biodegradation test was performed in a diastase-enzyme-containing solution with an activity of 480 KNU·g^−1^. A buffer solution (pH = 7.3) was prepared in a beaker by mixing 5 mL of 0.2 M acetic acid with 45 mL of 0.2 M sodium acetate solution. The biocomposites were submerged in the solution for 30 days in an incubator (New Brunswich Scientific) at 37 °C. The samples were taken every 5 days and rinsed thoroughly with distilled water before a conditioning step in an oven at 60 °C for 24 h.

In the weight loss measurement, each specimen was taken periodically and wiped and dried to a constant weight at 60 °C in a vacuum oven. The weight loss percentage was measured using an analytical balance, and calculated using Equation (2):

(2) $$\mathrm{Weight}\;\mathrm{loss}\;=\;\frac{W_{\mathrm{initial}}-W_{\mathrm{final}}}{W_{\mathrm{initial}}}\times \;100$$

where, W_initial_ and W_final_ stand for the weights of the biocomposites before and after the biodegradation test, respectively. The average value of the three measurements from each biocomposite was reported. 

## 3. Results and Discussion

### 3.1. Structural Analysis of Modified Chitosan

The OSL had a weight-average molecular weight (M_w_) of 3429, and a number-average molecular weight (Mn) of 2506. The polydispersity (M_w_/M_n_) of the OSL obtained was 1.37. These values were calculated from the chromatogram produced by the gel permeation chromatography. These results suggest that the extraction significantly ruptured the complex macromolecular structure of the lignin. A low polydispersity value indicates a narrow molecular weight distribution.

**Figure 1:**
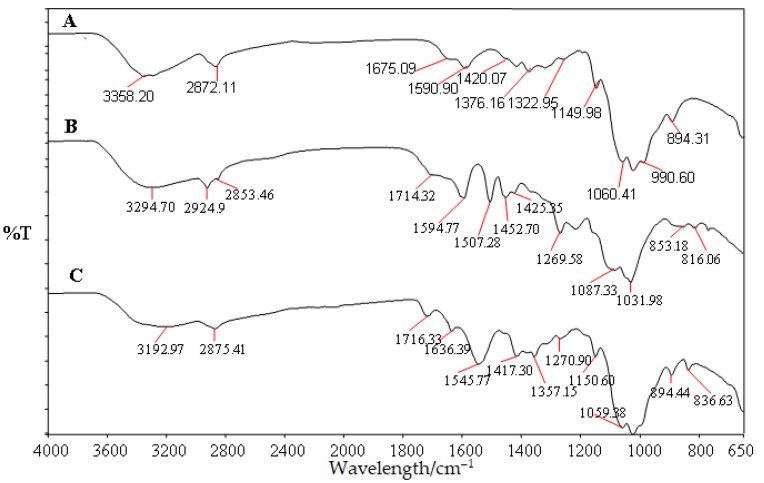
FTIR spectra of unmodified chitosan fiber and modified chitosan with PAA-g-OSL. (**A**) chitosan, (**B**) PAA-g-OSL modifying agent, (**C**) modified chitosan with PAA-g-OSL.

**Figure 2:**
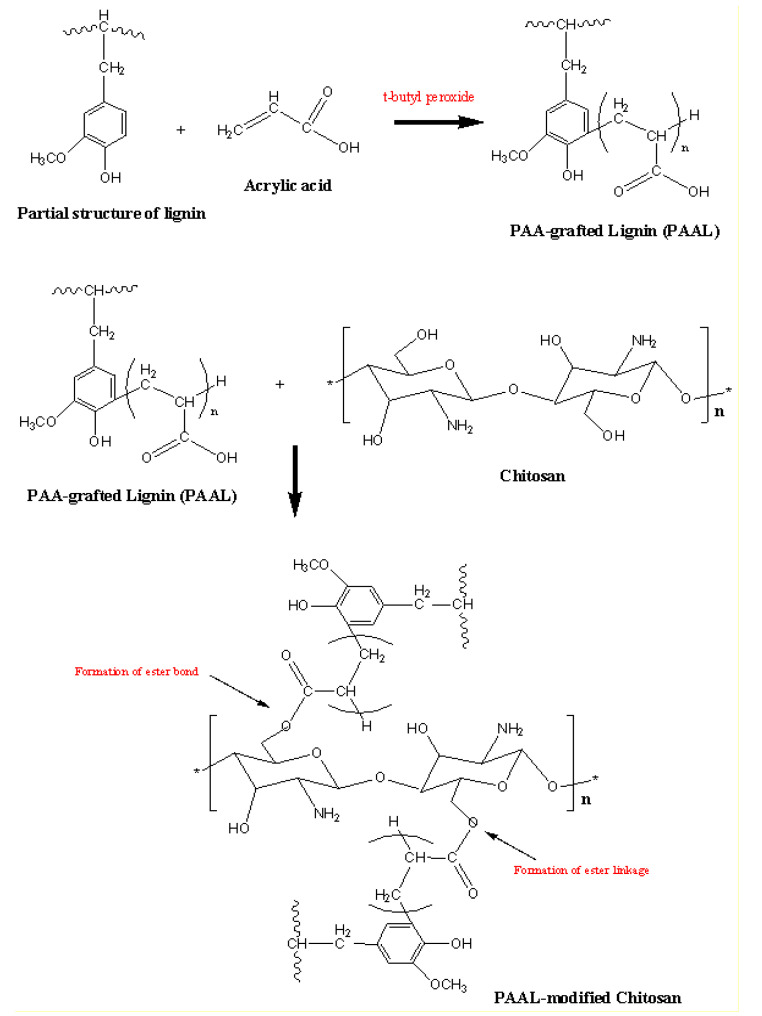
A possible schematic grafting reaction between OSL and acrylic acid, and the chemical modification of chitosan fiber.

Figure 1 shows FTIR spectra for unmodified chitosan fiber, and modified chitosan with PAA-g-OSL. The FTIR analysis was aimed at investigating the possibility of a chemical bonding formation between the PAA-g-OSL modifying agent and the chitosan fiber. The IR spectra belonging to unmodified chitosan demonstrate the existence of characteristic peaks at 3358 cm^−1^ (O–H stretch), 2872 cm^−1^ (C–H stretch), 1675 cm^−1^ (N–H bend), and 1590 cm^−1^ (C=O stretch). The differences between the absorption spectra of the modified and unmodified chitosan indicates an alteration in the chemical structure of chitosan upon the esterification process. The distinct change in the absorption spectrum, occurring on the peak between 1760–1712 cm^–1^ and 3400–3200 cm^–1^, was caused by the carboxyl groups (C–OH) of the modifying agent linked to the hydroxyl groups (–OH), chitosan-fiber-formed ester linkages (C–O–C), and the decrease of the content of chitosan’s hydroxyl groups. The formation of ester bonds (C–O–C) resulted in a decrease in hydrophilic moieties on the chitosan surface. The modified chitosan had less hydrophilicity (24.2% lower) than the unmodified chitosan. It was shown by a relatively lower intensity of the peak at 3292 cm^–1^ of the modified chitosan with PAA-g-OSL than that of the peak at 3358 cm^–1^ of unmodified chitosan. Figure 2 shows a possible schematic of the chemical reaction between chitosan fiber and esterified lignin.

### 3.2. Mechanical Properties of PLA/Chitosan Biocomposites

**Figure 3:**
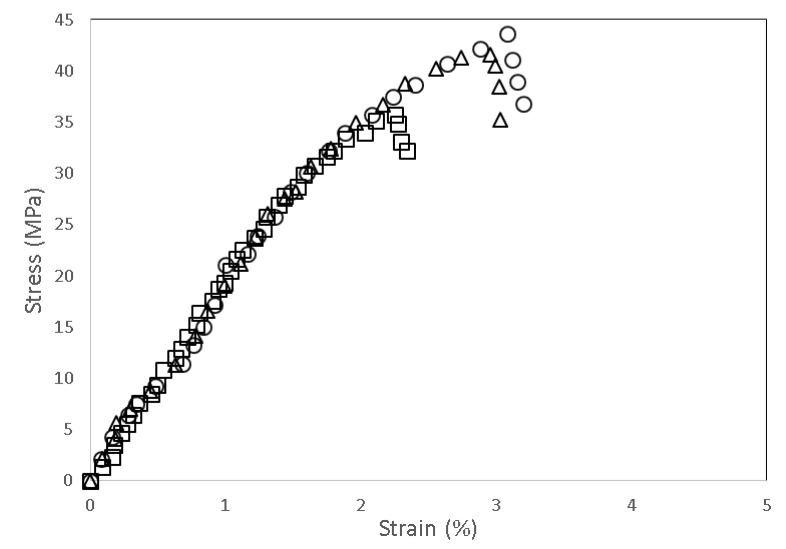
Stress–versus–strain traces of tensile testing for neat PLA (∆), unmodified PLA/chitosan biocomposite (□), and modified PLA/chitosan biocomposite with PAA-g-OSL (o). Chitosan content: 20 php.

Figure 3 shows typical stress–vs–strain traces obtained from the tensile tests for neat PLA, unmodified PLA/chitosan biocomposites, and modified PLA/chitosan biocomposites, respectively. All of the PLA biocomposites contained 20 php of chitosan fiber. All curves clearly show the same trend, where the incorporation of chitosan decreased the strength and strain of the PLA biocomposites, corresponding to poor interface interaction between the PLA and chitosan. Chitosan is a natural polymer that possesses polar hydrophilic properties, whereas PLA is a nonpolar hydrophobic polymer. The differences in the affinities and polarity diminished the interaction on the filler-matrix interface [34]. The modified PLA/chitosan biocomposite with PAA-g-OSL, on the other hand, demonstrated a greater strength than those of the neat PLA and the unmodified biocomposites, which indicates the enhanced interfacial adhesion upon the existence of PAA-g-OSL on the chitosan surface. Interestingly, the strain increased by 24.57% when compared with the unmodified biocomposite, reflecting the enhanced chain mobility of the PLA matrix within the biocomposite. Overall, it can be observed that the interfacial compatibility improves the composites’ strength. Although chemical modification had no effect on the stress–versus–strain trace character of the modified biocomposites, it did influence the measured strength at failure.

**Figure 4:**
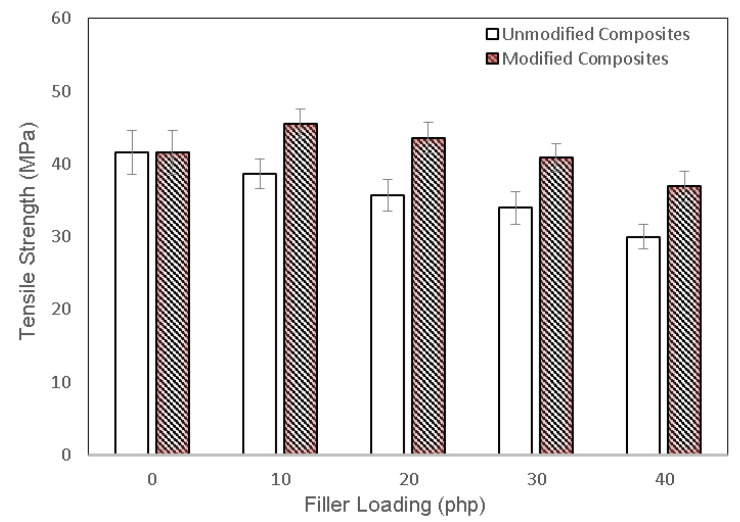
Effect of chitosan content and PAA-g-OSL on tensile strength of PLA/chitosan biocomposites.

The tensile strength of unmodified and modified PLA/chitosan biocomposites at various fiber loading are exhibited in Figure 4. The tensile strength of the PLA biocomposites was inversely proportional to the chitosan content. However, the modified biocomposites showed a higher tensile strength than that of the unmodified biocomposites at a similar chitosan content. The tensile strength of the modified biocomposites improved by 36.43% as compared with the unmodified ones, indicating the enhanced filler-matrix interfacial interaction. This enhanced strength was clearly attributed to the high reactivity of PAA-g-OSL, which effectively facilitated surface modification, resulting in improved interfacial adhesion.

**Figure 5:**
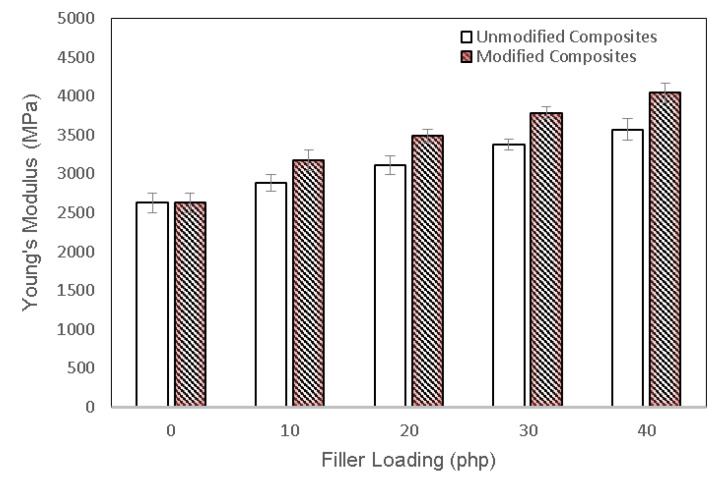
Young’s modulus of unmodified and modified PLA biocomposites at various chitosan content.

Figure 5 shows the Young’s modulus of unmodified and modified PLA biocomposites at different chitosan loading. The Young’s modulus of the PLA/chitosan biocomposites increased as the chitosan content increased. The dispersion of chitosan fiber within the PLA matrix clearly improved the composite’s stiffness, indicating the limited segmental molecular mobility of the PLA chain upon interacting with the fiber. The Young’s modulus of the modified biocomposites was remarkably higher than the unmodified biocomposites. It increased by 55.33% as compared with the unmodified ones. The rigid nature of the filler, the high crystallinity index, and the presence of the modifying agent played an essential role in the improvement of the Young’s modulus of the PLA biocomposites. The presence of esterified lignin improves the stiffness of the biocomposites by increasing the filler-matrix interfacial adhesion.

### 3.3. SEM Image Analysis

**Figure 6:**
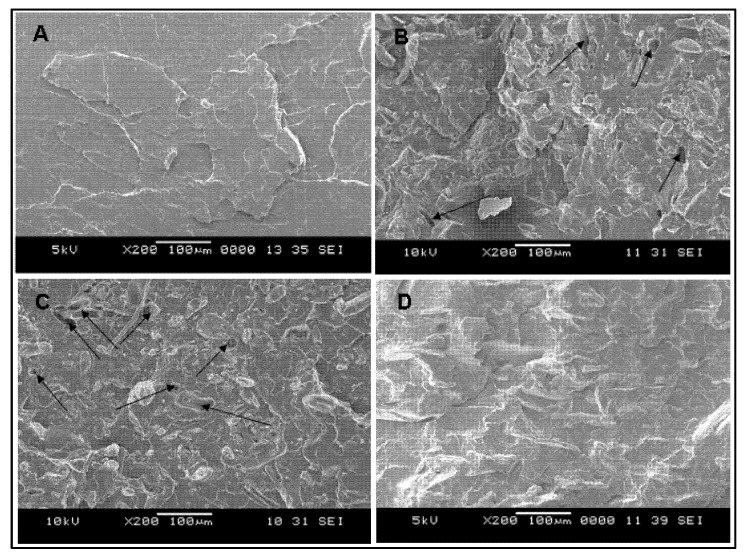
SEM micrographs of PLA/chitosan biocomposites, (**A**) neat PLA, (**B**) unmodified biocomposite (20 php), (**C**) unmodified biocomposite (40 php), (**D**) modified biocomposite (20 php). The arrows indicate the fiber pulled out traces.

Morphological images of the tensile fractured surfaces of the PLA matrix, unmodified PLA biocomposite containing 20 php and 40 php of chitosan fiber, and modified PLA composite filled with 40 php of chitosan fiber, are shown in Figure 6A–D, respectively. The surface of the PLA matrix was quite smooth, as seen in the SEM image (Figure 6A). A few voids, on the other hand, appeared on the fractured surface of the unmodified biocomposite (Figure 6B,C), exhibiting the fiber pulled out from the matrix phase. It pointed to the matrix’s poor wetting of the filler, which could lead to the breakage at the interface of the chitosan and the PLA matrix. The difference in the affinities between both components has resulted in weak interaction at the interface. Meanwhile, a relatively smooth surface with fewer voids was seen on the fracture surface of the modified biocomposite (Figure 6D). This was clearly related to a good wetting of the filler by the matrix since the PAA-g-OSL modifying agents substantially modified the chitosan surface to become less-hydrophilic, leading to improved interaction with the polymer matrix. As a result, the modified composites had significantly fewer detached filler traces from the matrix phase.

### 3.4. Thermal Properties of PLA/Chitosan Biocomposites

**Figure 7:**
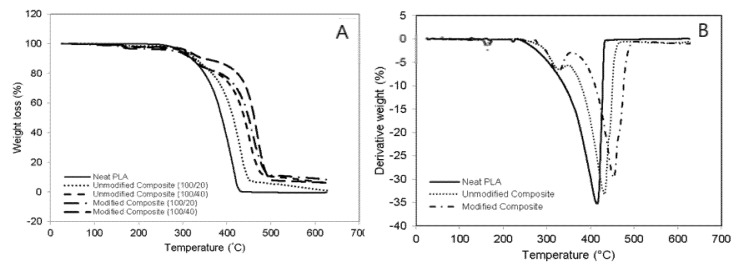
Effect of chitosan content and chemical modification on thermogravimetric properties of PLA/chitosan biocomposites. (**A**) weight loss versus temperature curves, (**B**) derivative thermogravimetry (DTG) curves.

Figure 7A,B show the TGA and DTG curves of neat PLA, unmodified PLA/chitosan biocomposites, and modified PLA/chitosan biocomposites, respectively. The thermal decomposition of neat PLA occurred over a one-stage process between 300 and 415 °C, leading to the formation of gaseous products. This degradation pathway was associated with the molecular structure of PLA that is composed of carbon–carbon linkages, where the degradation/depolymerisation occurs at the weak sites within the PLA backbone chain [35]. On the other hand, the PLA/chitosan biocomposites demonstrated three distinct stages of thermal decomposition. They were observed within the temperature ranges of 160–180 °C, 260–340 °C, and 400–490 °C, respectively. The similarity in the general patterns was exemplified by these typical thermograms. However, the biocomposites were indicated by their distinct temperature and weight losses. The first stage involved the release of typical strong hydrogen-bonded water and the evaporation of volatile compounds from the samples, while the second stage included the degradation and depolymerization of both the chitosan fiber and a modifying agent. The third stage could be attributed to the decomposition of char residue formed in the second stage.

The weight loss percentage for neat PLA, unmodified PLA/chitosan biocomposites, and modified PLA/chitosan biocomposites are tabulated in Table 3. An analysis of the weight loss data shows that 10% of the weight loss occurred at 328 °C for the unmodified biocomposites, and at 335 °C for the modified biocomposites. The temperature increased to 414 °C for the unmodified biocomposites, and to 440 °C for the modified biocomposites, when the weight loss was 50%. The obtained results clearly indicate that the modified biocomposites were more thermally stable than the unmodified ones. The attachment of the PAA-g-OSL modifying agent on the chitosan surface enabled it to perform as a thermal protective barrier for the biocomposites against thermo-decomposition.

Lignin is composed of a three-dimensional network of aromatic structures that can slow down the thermo-decomposition of polymeric materials. This, along with the complex heterogeneity of the chemical bonds in the cross-linked aromatic and aliphatic structures, is one of its primary defences against decomposition [36]. Furthermore, the data indicates that the modified biocomposites had higher thermal stability than the unmodified biocomposites. Despite the fact that chemical modification had no effect on the thermal decomposition mechanism of the PLA/chitosan biocomposites, the resulting different degradation profiles is indicative of the changing of the chemical structure and the thermal stability.

**Figure 8:**
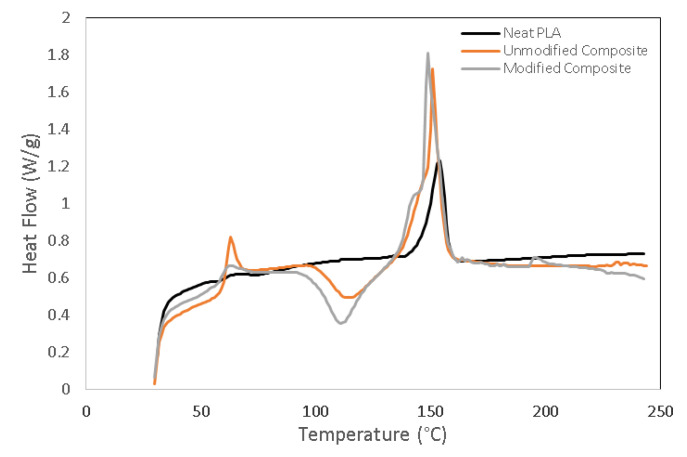
Differential scanning calorimetry (DSC) curves of unmodified and modified PLA/chitosan biocomposites at different fiber contents.

Figure 8 exhibits the DSC curves for neat PLA, unmodified PLA biocomposites, and modified PLA biocomposites, respectively. Table 4 provides the melting temperature (T_m_), the glass transition temperature (T_g_), crystallization temperature (T_c_), and the degree of crystallinity (*X*_c_) of all biocomposite categories. For all biocomposite categories, the endothermic peak was observed at a maximum temperature range of 150 to 155 °C. Neat PLA had a T_g_ of 57.6 °C, and a T_m_ of 152 °C. The addition of chitosan fiber increased both the T_g_ and T_m_ of neat PLA, while the crystallinity degree (*X*_c_) value slightly declined. This decreased crystallinity indicated that the addition of chitosan in PLA biocomposites apparently became an obstacle for the nuclei crystal growth of the PLA chain, causing the crystallization process to be hindered. [37]. In addition, a crystallization peak (T_c_) also appeared at a temperature of 115 °C, corresponding to the formation of the metastable crystalline phase within the biocomposites. 

On the other hand, a higher endothermic heat flow was observed for the modified biocomposites as compared to that for the unmodified biocomposites. Both the T_g_ and T_c_ of the modified biocomposites demonstrated a slight decrease; however, the *X*_c_ was obviously higher than that of the unmodified ones. It is thought that the presence of a PAA-g-OSL-based modifying agent improved the interfacial interaction between the chitosan and the PLA matrix, contributing significantly to the increased crystallinity of the modified biocomposites. It should be noted that the hydrophobic segment belonging to organosolv lignin may contribute to the effectiveness of the nucleation process, as well as the growth of the spherulitic structure in PLA biocomposites. The aromatic molecular structure and the chemical composition of organosolv lignin are expected as factors that can enhance the nucleation process. A previous study reported that some crystallization might occur when the fibers used contain sufficient quantities of lignin [38]. Nonetheless, the T_m_ did not show a significant change.

### 3.5. Weight Loss Analysis

**Figure 9:**
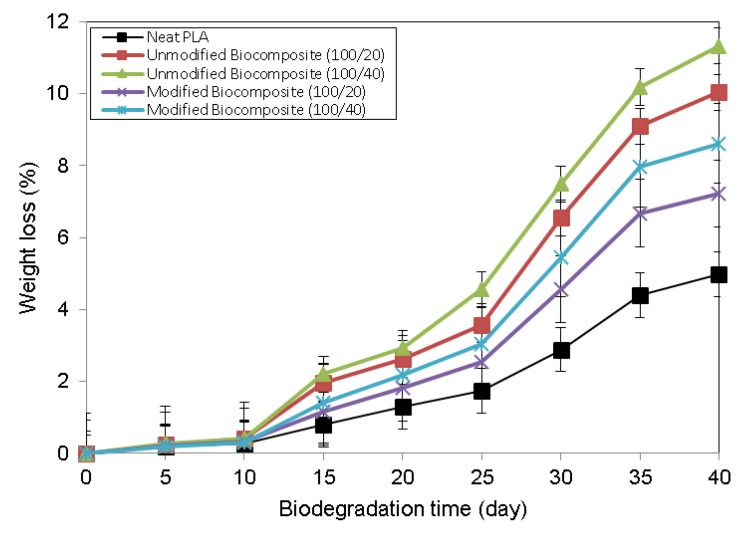
Percentage of weight loss of neat PLA, and unmodified and modified PLA/chitosan biocomposites, as a function biodegradation time.

Figure 9 exhibits the weight loss rate of neat PLA, unmodified PLA/chitosan biocomposites, and modified PLA/chitosan biocomposites as a function of the biodegradation days. As time passed, all the biocomposite categories degraded further, as evidenced by the increase in weight loss. The degradation of neat PLA was slower as compared to that of the two-biocomposite category. On the other hand, the addition of chitosan fiber apparently contributed to the increase in the biodegradation rate of the PLA biocomposites. The weight loss of the unmodified biocomposites was found to be about two-fold greater than that of the neat PLA, which confirmed the chitosan fiber dispersion within the PLA matrix and remarkably enhanced the degradation rate of the biocomposites. The chitosan fiber contributed to the high polarity of the biocomposites, resulting in decreased tensile strength. The improved biodegradation rate of the PLA/chitosan biocomposites was most likely because of the existence of the hydroxyl and amine groups belonging to chitosan, which, when dispersed in PLA, play a critical role in provoking the increasing hydrolytic degradation of the PLA main chain [39]. On the other hand, the biodegradation rates of the modified PLA/chitosan biocomposites were lower than that of the unmodified biocomposites, demonstrating that the chemical modification essentially enhanced the barrier performances of the composites and limited enzyme diffusion. The enhanced interfacial interaction between the PLA matrix and the chitosan fiber resulted from the PAA-g-OSL modifying effects, which decreased the area degraded through enzyme hydrolysis, leading to lower biodegradation rates.

One of the most important practical criteria for determining material degradation is the loss of mechanical properties. Table 5 provides tensile strength and the Young’s modulus of neat PLA, unmodified PLA/chitosan biocomposite, and modified PLA/chitosan biocomposite at a 0- and 30-day biodegradation time. Before immersion in the enzyme-containing solution, the tensile strength of neat PLA was observed to have decreased along with the addition of 20 php of chitosan fiber. On the other hand, the Young’s modulus improved. The presence of PAA-g-OSL on the chitosan surface contributed to the improvement in the tensile strength and the Young’s modulus of the modified biocomposites as compared to the unmodified biocomposite. The results show a decrease in the tensile strength and the Young’s modulus after 30 days of biodegradation. The action of the enzyme in the solution resulted in the apparent partial destruction of filler-matrix adhesion, void formation, and the subsequent random chain scission in the polymer chains, resulting in the decreased tensile properties.

## 4. Conclusions

PLA-chitosan biocomposites modified with PAA-g-OSL were prepared. The properties of the modified biocomposites were investigated and are briefly discussed. The obtained organosolv lignin had a low molecular weight, which resulted in a higher efficiency of chitosan modification of up to 21.3%. The PAA-g-OLS-based modifying agent significantly altered the chitosan surface, resulting in improved interfacial adhesion within the biocomposites. The mechanical strength of the modified biocomposites was greater than the mechanical strength of the unmodified biocomposites. The SEM images clearly show that the modified biocomposites had fewer voids on their tensile surface. The TGA and DSC results show that the modified biocomposites had improved thermal stability and crystallinity. Moreover, the modified biocomposites had a higher crystallinity degree than the unmodified biocomposites. Overall, the obtained results clearly suggest the advantages in utilizing grafted organosolv lignin-based modifying agents, and other lignin derivatives, in the manufacturing of natural fiber-reinforced biocomposite materials, thereby opening up the “door” to the potential for a renewable low-cost chemical reagent alternative to synthetic reagents.

## Figures and Tables

**Table 1:** Physical and chemical characteristics of chitosan.

Item	Specification	Test Method
Appearance	Off-white powder	Visual
Particle size	80 µm	Malvern Particle Size Analyzer
Solubility of 1% chitosan in 1% acetic acid	>99.0%	Dissolution & Filtration
Viscosity	150–200 m Pa·s	Ubbelohde Viscometer
Moisture content	<10.0%	Infra-red drying
Ash content	<1.0%	Incineration

**Table 2:** Formulation of unmodified and modified PLA biocomposites at different chitosan contents.

Materials	Unmodified Biocomposites	Modified Biocomposites with PAAL
Polylactic acid (PLA) (php)	100	100
Chitosan (php)	0, 10, 20, 30, 40	10, 20, 30, 40
PAA-g-OSL (PAAL) (wt%)	-	3

php = part per hundred of polymer.

**Table 3:** Weight loss percentage of unmodified and modified PLA biocomposites containing chitosan fiber at different temperatures.

Temperature (°C)	Neat PLA	Unmodified Biocomposites		Modified Biocomposites	
		20 php	40 php	20 php	40 php
100–150	0.002	0.039	0.277	0.342	0.600
150–200	0.016	0.271	0.555	0.464	0.773
200–250	0.150	1.017	1.894	0.943	1.561
250–300	0.476	0.157	0.212	0.428	0.673
300–350	4.993	0.495	1.262	2.102	3.419
350–400	14.813	4.540	5.080	3.988	2.005
400–450	43.150	13.539	13.105	10.197	9.663
450–500	36.400	40.499	36.512	38.738	31.997
500–550	0	33.153	32.855	32.834	29.461
550–600	0	1.494	1.428	1.359	9.302
600–630	0	1.886	1.692	0.348	0.674
Total	100	97.090	94.872	91.743	90.128

php = part per hundred of polymer.

**Table 4:** DSC data obtained for unmodified and modified PLA/chitosan biocomposites.

Biocomposites	T_g_ (°C)	T_c_(°C)	T_m_ (°C)	*X*_c_ (%)
Neat PLA	57.6	-	152.0(0.2)	32.74
PLA/chitosan: 100/20 php (unmodified)	63.4	117.7	151.3(0.3)	27.41
PLA/chitosan: 100/40 php (unmodified)	62.5	117.2	151.1(0.1)	24.25
PLA/chitosan: 100/20 php (modified)	61.3	115.3	150.4(0.5)	39.42
PLA/chitosan: 100/40 php (modified)	61.2	114.5	150.2(0.3)	33.80

T_g_: glass transition temperature; T_c_: crystallization temperature; T_m_: melting temperature; *X*_c_: crystallinity degree; php: parts per hundred of polymer.

**Table 5:** Tensile strength and Young’s modulus of neat PLA, PLA/chitosan biocomposites, with and without treatment, at 0- and 30-day biodegradation times.

Biocomposites	0 Day		30 Day	
	Tensile Strength (MPa)	Young’s Modulus (MPa)	Tensile Strength (MPa)	Young’s Modulus (MPa)
Neat PLA	42.82	2674.68	35.67	2476.43
PLA/chitosan: 100/20 (unmodified)	37.74	3158.97	28.84	2847.91
PLA/chitosan: 100/40 (unmodified)	31.81	4125.86	25.65	3789.36
PLA/chitosan: 100/20 (modified)	45.64	3567.45	40.27	3254.29
PLA/chitosan: 100/40 (modified)	38.72	4180.87	32.85	3810.38

## Data Availability

Not applicable.
